# A stable spin-structure found in a 3-body system with spin-3 cold atoms and its role in N-body condensates

**DOI:** 10.1038/s41598-021-81133-7

**Published:** 2021-01-19

**Authors:** Y. M. Liu, Y. Z. He, C. G. Bao

**Affiliations:** 1 Department of physics, Shaoguan University, Shaoguan, 510205 People’s Republic of China; 2 School of Physics, Sun Yat-Sen University, Guangzhou, 510275 People’s Republic of China; 3 State Key Laboratory of Theoretical Physics, Institute of Theoretical Physics, Chinese Academy of Sciences, Beijing, 100190 People’s Republic of China

**Keywords:** Bose-Einstein condensates, Theoretical physics

## Abstract

We have found a stable spin-structure of N $=$ 3 system in which three spin-3 atoms are trapped and coupled to total spin S $=$ 3. We have proved that a pair of this structure is nearly an exact solution for N $=$ 6 systems in a very broad district in the parameter-space. Comparing with the well-known singlet pairs, this pair is a more promising candidate to serve as a building block for large N systems with spin-3 atoms. This is because the spin-structure of the latter can be modified depending on the interactions to reduce the energy while the singlet pairs can not. In fact, we have proved that, for a specific set of strengths (a point in the parameter-space) the product state based on this pair is an exact solution of the N-body Hamiltonian. Thus, in the neighborhood of this point, the product state will appear as an approximate solution. However, how broad this neighborhood would be remains to be clarified.

## Introduction

It is well known that the study of the Bose–Einstein condensates as an artificial matter is important in the academic sense and for practical applications. In particular, since the realization of optical trapping^1–9^, the study of the spinor condensates has become a hot topic. When the temperature is very low, the spatial degrees of freedom are nearly frozen and the spin-degrees of freedom play essential roles. For this case, the understanding of the spin-structures is crucial. Various structures (phases) have already been found. For condensates with spin-1 atoms, the ground state (g.s.) may have the ferro-phase (*f*-phase, where all the spins are aligned along the same direction) and polar-phase (*p*-phase, where all the spins are two-by-two couples to zero and form the singlet-pairs). For spin-2 condensates, the *f*-phase, *p*-phase, together with the cyclic-phase (*c*-phase, where the g.s. is nearly a product-state of the triplets, in each triplet the three spin-2 atoms are coupled to zero) are found^8,10–17^. There are also studies for spin-3 condensates, where the structures appear to be complicated^18–24^.

Due to the progress in technology, it is possible to put only a few atoms in a trap. In the theoretical aspect, instead of using mean-field theory, exact solutions for few-body systems can be obtained. The knowledge extracted from few-body systems would be a complement to those from many-body theories. Furthermore, these few-body cold systems might be more suitable for realistic applications because they can be more precisely controlled.

The present paper is dedicated to the study of spin-3 cold atoms. The main purpose is to find out some stable constituents from few-body systems and to evaluate their potential for serving as a building block for large *N* systems.

## Exact solutions of the 3-body Schrödinger equation

Let three spin-3 atoms (say, Cr, Mo, Sn, Pu) be confined in an optical trap. It is assumed that the temperature is so low and the binding is so strong that all the particles have condensed to the same spatial state $\phi (\mathbf {r})$ with zero orbital angular momentum. The state $\phi (\mathbf {r})$ is most favorable for binding, the excitation of this state is not considered. When all the spatial degrees of freedom have been frozen, only the spin-degrees of freedom are necessary to be considered. Then, the Hamiltonian can be written as

1 $$\begin{aligned} H&= \sum _{i<j}V_{ij}, \end{aligned}$$

2 $$\begin{aligned} V_{ij}&= \sum _{\lambda }g_{\lambda }P_{\lambda }^{ij}, \end{aligned}$$

where *i* (*j*) denotes the particle 1 to partical 3. $\lambda$=0, 2, 4, and 6 is the coupled spin of two particles, $P_{\lambda }^{ij}$ is the projector to the $\lambda$-channel. $g_{\lambda }$ is the weighted strength where a factor $\int \phi ^4d\mathbf {r}$ is contained. This factor embodies the effect of the spatial wave function $\phi$ on the spin-structures. Since $\phi$ has orbital angular momentum zero, the orbital-spin coupling is suppressed and therefore is neglected. The dipole-dipole (*d*-*d*) coupling between a pair of atoms is relatively weak (for $^{52}$Cr as an example, the strength of the *d*-*d* coupling $c_{dd}=0.004g_6$), therefore it is also neglected. In fact, the calculation in^19^ demonstrates that the g.s. of $^{52}$Cr does not seem to depend on the *d*–*d* coupling.

An important feature of *H* is the conservation of the total spin *S*. Due to symmetry constraint, *S* is allowed to be equal to 1, 3, 4 to 7, and 9. The corresponding total spin-states $\psi _S$ are studied and given below.

### The case $S\ne 3$

In this case the multiplicity of $\psi _S$ is one. It can be written as $\psi _S=\mathfrak {P}((\chi \chi )_{\lambda }\chi )_S$, where $\chi$ denotes the spin-state of a spin-3 atom, the first two spin-states are coupled to $\lambda$, then the three are coupled to *S*. $\mathfrak {P}$ is the operator for symmetrization and normalization. $\lambda$ is an even number and $|3-S|\le \lambda \le 3+S$. Since the multiplicity of $\psi _S$ is one, $\lambda$ can be arbitrary chosen (i.e., different choices of $\lambda$ lead to the same $\psi _S$).

By re-coupling the three spins, $\psi _S$ can be rewritten as

3 $$\begin{aligned} \psi _S=\sum _{\eta }C_{\lambda \eta }^S((\chi (1)\chi (2))_{\eta }\chi (3))_{SM}, \end{aligned}$$

where $\eta$ should be even,

4 $$\begin{aligned} C_{\lambda \eta }^S=(\delta _{\lambda \eta }-2(-1)^S\sqrt{(2\lambda +1)(2\eta +1)}W(33S3;\lambda \eta ))/\mathfrak {N}, \end{aligned}$$

where the W coefficient of Racah has been introduced, the constant $\mathfrak {N}$ is introduced to assure $\sum _{\eta }(C_{\lambda \eta }^S)^2=1$. In Eq. (3) particles 1 and 2 have been extracted. Note that, due to the symmetry, the labels 1, 2, and 3 at the right side of Eq. (3) can be replaced by any other permutations of the three numbers. Making use of this feature and the multiplicity of $\psi _S$ we can prove

5 $$\begin{aligned} H\psi _S&=E_S\psi _S, \end{aligned}$$

6 $$\begin{aligned} E_S&=3\sum _{\eta }g_{\eta }(C_{\lambda \eta }^S)^2. \end{aligned}$$

Note that $\psi _S$ as an eigen-state of *H* does not depend on $\{g_{\eta }\}$ but simply on symmetry. Since the choice of $\lambda$ is irrelevant, $C_{\lambda \eta }^S$ can be rewritten as $C_{\eta }^S$ in short.

Inserting Eq. (3) into the equation $\langle \psi _S|\psi _S\rangle =1$, we can deduce that the probability of an atom lying at the $\mu$-component ($-3\le \mu \le 3$) is

7 $$\begin{aligned} P_{\mu }^{SM}=\sum _{\eta }(C_{\eta ,M-\mu ;3,\mu }^{SM}C_{\eta }^S)^2, \end{aligned}$$

The probability that two atoms are lying at $\mu$ and $\nu$, respectively, are

8 $$\begin{aligned} P_{\mu \nu }^{SM}=\sum _{\eta }(C_{\eta ,M-\mu ;3,\mu }^{SM}C_{3,\nu ;3,M-\mu -\nu }^{\eta ,M-\mu }C_{\eta }^S)^2. \end{aligned}$$

Furthermore, from Eq. (3), $(C_{\eta }^S)^2$ is the probability of a pair of particles being coupled to $\eta$, This explains the origin of $E_S$ as given in Eq. (6).

### The case $S=3$

When $S=3$ the multiplicity is two. The two spin-eigen-states are denoted as $\psi _{3k}$ ($k=1$ for the lower and 2 for the higher). They can be expanded as

9 $$\begin{aligned} \psi _{3k}=\sum _{\eta }C_{\eta }^{3k}((\chi (1)\chi (2))_{\eta }\chi (3))_3, \end{aligned}$$

where $C_{\eta }^{3k}$ depends on $\{g_{\eta }\}$. $\psi _{3k}$ can also be analytically obtained as shown in Suppl. Appendix 1. The associated eigen-energy $E_{3k}=3\sum _{\eta }g_{\eta }(C_{\eta }^{3k})^2$.

Up to now, with the freezing of the spatial degrees of freedom, all the eigen-states and eigen energies of the $N=3$ system have been found. each specifies a kind of spin-structure. It is evident that the multiplicity is very important to the spin-structures of few-body systems. When the multiplicity is one, the structure is irrelevant to dynamics but completely determined by symmetry. Whereas for those spin-states with multiplicity $\ge 2$, they can be modified by adjusting the strengths of interaction. These states are noticeable.

## Phase-diagrams for $N=3$ systems

**Figure 1:**
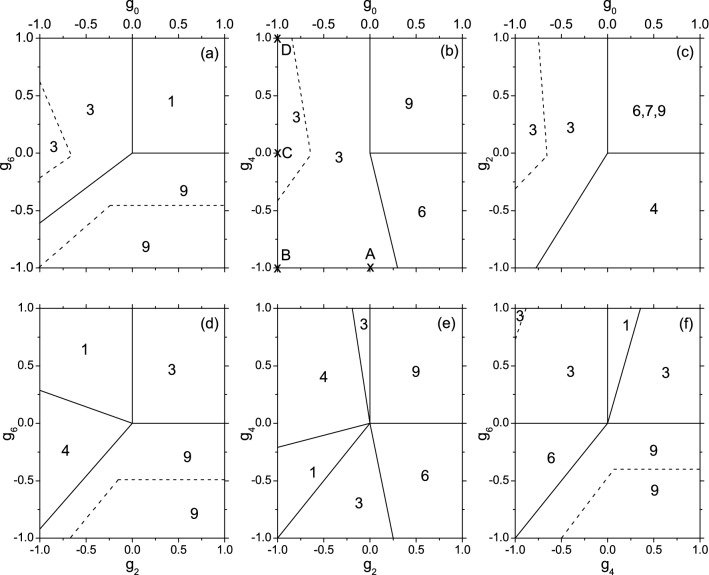
The phase-diagram of a 3-body trapped system with spin-3 cold atoms. In each panel two $g_{\eta }$ are chosen to serve as the ordinate and abscissa. The values of the other two $g_{\eta }$ are given at zero. The phase of the g.s. is specified by the total spin *S* marked inside the related zone. The district bound by the dotted lines has $E_{gap}\ge 0.8$.

We will neglect the higher state $\psi _{3,2}$, then $\psi _{3,1}$ is rewritten as $\psi _3$. Among the seven $\psi _S$ ($S=1$ to 9, except 2 and 8), the one having the lowest energy is the g.s. The phase of the g.s. can be specified by *S*. The phase-diagram is plotted in Fig.1 where the variation of the phase against $\{g_{\eta }\}$ is shown. For any sets of $\{g_{\eta }\}$, we find out the two, say, $g_a$ and $g_b$, having the smallest $|g_a-g_b|$. Then, as an approximation, $g_a=g_b=\frac{g_a+g_b}{2}$ is assumed. Note that: (i) If all the $g_{\eta }$ are shifted by a common value, then the total energy will shift accordingly but the spin-structures will remain unchanged. (ii) If the unit of energy is changed, the spin-structures will remain unchanged. Thus, we adopt a shift so that $\frac{g_a+g_b}{2}$ is shifted to zero, and we adopt a new unit so that the scope $-1\rightarrow 1$ is sufficient. Then, the 4-dimensional complicated phase-diagram can be replaced by six 2-dimensional diagrams. They are sufficient to reveal the qualitative features. In order to understand the stability of the g.s., the energy gap $E_{gap}$ (the energy difference between the first excited state and the g.s.) has been calculated. The districts with $E_{gap}\ge 0.8$ are marked, in which the g.s. is relatively more stable (the choice 0.8 is quite arbitrary, it is so chosen that a smaller part of the whole zone appears as a relatively more stable zone.).

The feature of a spin-structure is inherent in the coefficients $\{C_{\eta }^S\}$ which arises from symmetry constraint. Their squares are listed in Table 1. It is Noted that these coefficients does not depend on $\{g_{\eta }\}$ except those for $S=3$ states. In the latter case four sets of $\{g_{\eta }\}$ are chosen as examples, they are associated with the points A to D marked in Fig. 1b.

**Table 1:** The squares of the coefficients $(C_\eta ^S)^2$ for the eigen-spin-states of the 3-body system. 3A to 3D are for the $S=3$ states with the parameters given at the points A to D marked in Fig. 1b.

*S*	$(C_0^S)^2$	$(C_2^S)^2$	$(C_4^S)^2$	$(C_6^S)^2$
1	0	0.524	0.476	0
3A	0.108	0.136	0.753	0.003
3B	0.234	0.031	0.687	0.049
3C	0.429	0.106	0.190	0.275
3D	0.380	0.263	0.025	0.331
4	0	0.611	0.061	0.328
5	0	0.413	0.234	0.353
6	0	0	0.727	0.273
7	0	0	0.515	0.485
9	0	0	0	1

From this table we see that

- Since $\psi _9$ has $C_6^9=1$, the contribution from $g_6$ is maximized. Therefore, when $g_6$ is the smallest (most negative), $S=9$ is the best choice. This leads to the *f*-phase. The appearance of this phase in large *N* systems has been well known.
- When $g_0$ is the smallest, due to all $C_0^S=0$ if $S\ne 3$, the $S=3$ is the best choice.
- When $g_4$ and $g_6$ both are smaller (this happens in the up-right corner of Fig. 1c and the down-left corner of Fig. 1f), the states with a larger $(C_4^S)^2$ and $(C_6^S)^2$ will be lower. Therefore the candidates of the g.s. are those with $S=6$, 7, and 9. For them and from Eq. (6), we have $E_S=g_6+(C_4^S)^2(g_4-g_6)$, where $(C_4^S)^2=0.727$, 0.515, and 0, respectively, for $S=6$, 7, and 9. Therefore, when $g_4<g_6$, the best choice is $S=6$ because $(C_4^6)^2$ is the largest. Whereas when $g_4>g_6$, the best choice is $S=9$ because $(C_4^9)^2$=0. In Fig. 1f the boundary separating the $S=6$ and 9 zones has $E_6-E_9=0$. This leads to $g_4-g_6=0$. In fact, once $g_4=g_6$, the three states with $S=6$, 7, and 9 are degenerate as shown in Fig. 1c.
- When $$g_2$$ and $$g_6$$ are smaller (the up-right corner of Fig. 1b and the down-left corner of Fig. 1d), the unfavorable contribution from $$g_0$$ and $$g_4 $$ would be minimized in $$\psi _4$$ and $$\psi _9$$ because $$(C_0^S)^2$$ and $$(C_4^S)^2$$ are zero or much smaller if $$S=4$$ and 9. In Fig. 1b, the up-right corner has $$E_4-E_9=0.061g_4>0$$. Thus, the zone has $$S=9$$. In Fig.1d, the down-left corner has $$E_4-E_9=0.611g_2-0.672g_6$$. Therefore, the zone will have $$S=4(9)$$ if $$g_2<(>)1.1g_6$$. The boundary separating the S=4 and 9 zones has $$g_2=1.1g_6$$.

Similarly, all the boundaries can be analytically explained. It is reminded that all the spin-structures with $S\ne 3$ are fixed by symmetry constraint. However, $\psi _3$ will change against $\{g_{\eta }\}$. For examples, when the point A in Fig. 1b is shifted to B, the g.s. is changed from $\psi _{3A}$ to $\psi _{3B}$ (refer to the second and third rows of Table 1). The shift implies a decrease of $g_0$ and accordingly an increase of $(C_0^S)^2$. In this way, the g.s. energy is reduced. Similarly, the shift B$\rightarrow$C$\rightarrow$D implies an increase of $g_4$ and accordingly a decrease in $(C_4^S)^2$, etc..

For $^{52}$Cr, when the strength of $g_6$ is used as an energy unit, then $g_6=1$, $g_4=0.517$, $g_2=-0.063$. By an exact numerical calculation on this $N=3$ system, we found a critical value $g_{crit}=-0.3$. When $g_0<g_{grit}$ the g.s. has $S=3$, whereas when $g_0>g_{grit}$, $S=1$. This information would be helpful for identifying $g_0$. This realistic case is qualitatively similar to the upper part of Fig. 1a. Where, when $g_0$ increases from -1$\rightarrow$1 along the upper boundary, *S* transits from 3$\rightarrow 1$ at the critical value $g_{crit}=0$.

We found that the more stable districts (bound by the dot line) either have $S=9$ or $S=3$. In the former all the spins are lying along the same direction. In the latter the spin-structure depends on $\{g_{\eta }\}$. We found from Fig. 1a–c that once $g_2=g_4=g_6=0$ and $g_0=-1$, $E_{gap}$ of $\psi _3$ will arrive at its maximum 1.2857 (say, at the point C of Fig. 1b). At the maximum the probabilities extracted from $\psi _3$ (refer to Eqs. (7) and 8)) are $P_3^{3,3}=0.481$, $P_{-3}^{3,3}=0.148$, while all the other $P_{\mu }^{3,3}=0.074$. It implies that, when $M=S$ is chosen and therefore *S* is essentially lying along the *Z*-axis, the spins are mostly lying along the $\pm Z$-axis. When one spin is given at $\mu =3$, we have $P_{3,\nu }^{3,3}/P_3^{3,3}=0.308$ (if $\nu =\pm 3$) or 0.077 (if $\nu \ne \pm 3$). This leads to an intuitive picture, namely, two spins are mostly lying along *S* while the third lying reversely. It is interesting to ask whether this relatively more stable structure $\psi _3$, in addition to $\psi _9$, would play a role as a building block in large *N* systems. To reply, we first go to $N=6$ system.

## Phase-diagrams for $N=6$ systems

**Figure 2:**
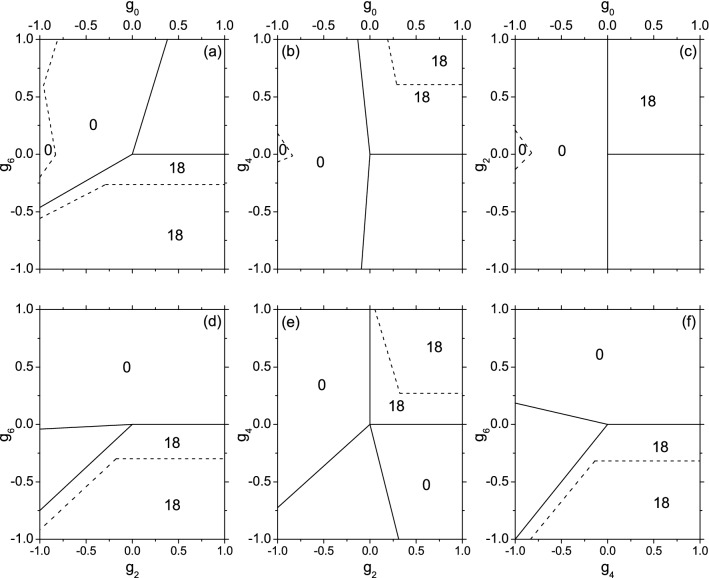
The phase diagrams for $N=6$ systems (refer to Fig. 1). Only the zones with $S=0$ and 18 are marked.

When $N=6$, we do not have analytical solutions. Instead, the solutions are obtained via a diagonalization of the Hamiltonian against the Fock-states as basis-states. The resultant phase-diagrams are given in Fig. 2.

We found that the zones with $S=18$ in Fig. 2 and those with $S=9$ in Fig. 1 overlap nearly. It implies that the *f*-phase would emerge in both systems under similar condition of $\{g_{\eta }\}$, i.e., $g_6$ alone is the smallest one (most negative), or $g_6$ and $g_x$ ($x=0$, 2, or 4) are both smaller. In the latter case there is a competition, say, in the down-left corner of Fig. 1d, $g_6<g_2/1.1$ is required to assure the *f*-phase. while in Fig. 2d a similar condition is found. Therefore, it is expected that the *f*-phase would emerge in large *N* systems under similar conditions. But this remains to be checked.

**Figure 3:**
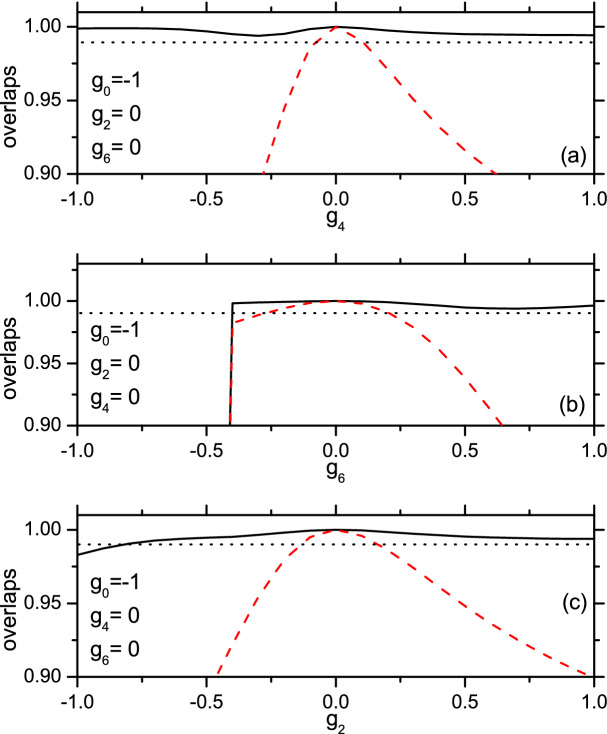
The overlaps $\langle \Psi _0|\mathfrak {P}(\psi _3\psi _3)_0\rangle$ (solid line) and $\langle \Psi _0|\mathfrak {P}(\chi \chi )_0^3\rangle$ (dashed line) against $g_4$, $g_6$, or $g_2$. In Fig. 3a–c, The varying strength is moving up along the left boundary of Fig.2b,a,c, respectively. The dotted horizontal line marks the value 0.99.

Let the $S=0$ exact eigen-states be denoted as $\Psi _0$, its multiplicity is 3. Thus, $\Psi _0$ has three kinds of spin-structures. It turns out that the zones with $S=0$ in Fig.2 are larger but include those with $S=3$ in Fig. 1. It implies that at least a kind of $\Psi _0$ might contain $\psi _3$ as a constituent. To clarify, we calculate the overlap $\langle \Psi _0|\mathfrak {P}(\psi _3\psi _3)_0\rangle$ (where the two $\psi _3$ states are coupled to zero) as shown in Fig. 3. Let $(\chi \chi )_0$ denote a singlet-pair (two atoms are coupled to zero). The overlap $\langle \Psi _0|\mathfrak {P}(\chi \chi )_0^3\rangle$ has also been given in Fig. 3 for a comparison.

Figure 3 demonstrates that, in a rather broad scope of the parameters, the overlap $\langle \Psi _0|\mathfrak {P}(\psi _3\psi _3)_0\rangle$ is $\ge 0.995$. In this case the g.s. can be nearly exactly described by $\mathfrak {P}(\psi _3\psi _3)_0$. Whereas $\langle \Psi _0|\mathfrak {P}(\chi \chi )_0^3\rangle$ is in general not close to 1 except in a very narrow district around the point with $g_0=-1$ and $g_2=g_4=g_6=0$. At the point both overlaps are equal to 1. In this particular case both $\mathfrak {P}(\psi _3\psi _3)_0$ and $\mathfrak {P}(\chi \chi )_0^3$ are identical to the exact solution. A distinguished feature of $\mathfrak {P}(\psi _3\psi _3)_0$ is its flexibility against the strengths, i.e., it can be adjusted to reduce the energy while $\mathfrak {P}(\chi \chi )_0^3$ cannot. This explains why the former surpasses the latter. Thus the former would be a better candidate of building block.

## Large *N* systems and final remarks

At the point in the parameter-space with $g_0<g_2=g_4=g_6$ (this set is equivalent to $g_2=g_4=g_6=0$ and $g_0$ is negative), we can prove $\mathfrak {P}(\psi _3\psi _3)_0^K=\mathfrak {P}(\chi \chi )_0^{3K}$ (if $N=6K$) or $\mathfrak {P}(\psi _3\psi _3)_0^K\psi _3=\mathfrak {P}(\chi \chi )_0^{3K+1}\chi$ (if $N=6K+3$). In particular, all of them are exact solutions of the Hamiltonian. The proof is given in Suppl. Appendix 2. This fact implies that these product states of the building blocks would be good approximate solutions for the g.s. at least in the neighboring district around the point. Due to the flexibility of the building block $(\psi _3\psi _3)_0$, the product state based on $(\psi _3\psi _3)_0$ is expected to be valid in a much broader district than that based on $(\chi \chi )_0$ as shown in Fig. 3 . Thus the pair $(\psi _3\psi _3)_0$ is superior than the singlet pair to serve as a building block for large *N* system.

In conclusion, we have studied the features of the spin-states of the $N=3$ system. We found that, in addition to the *f*-phase, where all the three spins are lying along the same direction, $\psi _3$ is also very stable, where two spins are essentially lying along the same direction while the third lying reversely. In particular, the $(\psi _3\psi _3)_0$ pair has been proved to be a nearly exact eigen-states for $N=6$ systems in a rather broad sub-space in the parameter space. The $(\psi _3\psi _3)_0$ pair is a promising candidate, superior to the singlet pair $(\chi \chi )_0$, for serving as a building block for *N*-body systems. Although the product state based on $(\psi _3\psi _3)_0$ has been proved to be an exact solution at the point with $g_0<g_2=g_4=g_6$, how broad is the district around the point in which the product state could be considered as a good approximation remains to be clarified.

In this paper a stable sub-structure has been extracted from $N=3$ system. It is likely that stable spin structures might also exist in $N\ge 4$ systems. For an example, the $S=4$ state of $N=4$ system with spin-3 atoms has multiplicity 3, thus this state has a better flexibility (the ability to modify its structure to reduce the energy). Therefore, it might serve as a building block for large *N* systems when the strengths are given in a specific region. Nonetheless, this is only a presumption. The role of the stable sub-structures from $N\ge 4$ systems remains to be clarified.

## Supplementary information

Supplementary information.
