# Supplementary material for: A stable spin-structure found in a 3-body system with spin-3 cold atoms and its role in N-body condensates
Source: Sci Rep. 2021 Jan 19;11:1792. doi: 10.1038/s41598-021-81133-7 (PMC7815769; doi:10.1038/s41598-021-81133-7)
Supplement: Supplementary file 1 — Supplementary information. [file 41598_2021_81133_MOESM1_ESM.pdf]

A stable spin-structure found in a 3-body system with spin-3 cold atoms and its role in N-body condensates

Y. M. Liu, Y. Z. He, and C. G. Bao

Appendix 1, The analytical forms of ψ_{3k}

Since the $S=3$ states have multiplicity two, the eigen-states can be obtained via a diagonalization of H in a space expanded by $\varphi_\lambda \equiv \mathfrak{P}((\chi\chi)_\lambda\chi)_3$ and $\varphi_{\lambda'} \equiv \mathfrak{P}((\chi\chi)_{\lambda'}\chi)_3$, where $\lambda \neq \lambda'$ are any two from 0, 2, 4, and 6. Different choices will lead to the same result. By re-coupling the spins, these states can be expanded as

$$\varphi_\lambda = \sum_\eta C^3_{\lambda\eta}((\chi(1)\chi(2))_\eta\chi(3))_3, \tag{1}$$

where $C^3_{\lambda\eta}$ are given in eq.(4) with $S=3$.

Let $\lambda=0$ and λ' =6. We define further

$$\varphi_q = \frac{\langle\varphi_0|\varphi_6\rangle}{\sqrt{1-\langle\varphi_0|\varphi_6\rangle^2}}\varphi_0 - \frac{1}{\sqrt{1-\langle\varphi_0|\varphi_6\rangle^2}}\varphi_6 \equiv \sum_\eta C^3_{q\eta}((\chi(1)\chi(2))_\eta\chi(3))_3, \tag{2}$$

One can prove that φ_q and φ_0 are orthogonal and can be used as basis-states for the diagonalization of H. Let $H_{00} = 3\sum_\eta (C^3_{0\eta})^2 g_\eta$, $H_{qq} = 3\sum_\eta (C^3_{q\eta})^2 g_\eta$, $H_{0q} = 3\sum_\eta C^3_{0\eta}C^3_{q\eta}g_\eta$. Then, the eigen-energies are

$$E_{3k} = \frac{1}{2}\{H_{00}+H_{qq} \pm \sqrt{(H_{00}+H_{qq})^2 - 4(H_{00}H_{qq}-H^2_{0q})}\}, \tag{3}$$

where the minus (plus) sign in $\pm$ is for $k=1$ (2). The eigen-state are

$$\psi_{3k} = \frac{1}{\sqrt{H^2_{0q}+(E_{3k}-H_{00})^2}}(H_{0q}\varphi_0 + (E_{3k}-H_{00})\varphi_q). \tag{4}$$

It is noted that the spin-structures would remain unchanged if all the $\{g_\eta\}$ are shifted by the same value. Thus, the case $g_0 < g_2 = g_4 = g_6$ is equivalent to the case $g_0 = -|g|$ and $g_2 = g_4 = g_6 = 0$. The latter leads to $H_{qq} = 0$ and $H_{0q} = 0$. Accordingly, $E_{3,1} = H_{00}$ and $\psi_{3,1} = \varphi_0$; and $E_{3,2} = 0$ and $\psi_{3,2} = \varphi_q$.

Appendix 2: An exact solution of large N systems based on $(\psi_3\psi_3)_0$ as a building block

We first assume that N is odd and we consider the symmetrized and normalized total spin-state

$$\Psi = \mathfrak{P}(\psi_3\psi_3)_0^K\psi_3, \tag{5}$$

where $K=(N-3)/6$.

When $g_0 < g_2 = g_4 = g_6 = 0$, we know from Appendix 1 that $\psi_3 = \varphi_0 = \mathfrak{P}(\chi\chi)_0\chi$. Accordingly, $\mathfrak{P}(\psi_3\psi_3)_0 = \mathfrak{P}(\chi\chi)_0^3$. Let $\mathfrak{P} \equiv \zeta\mathscr{P}$, where the coefficient ζ is for the normalization and $\mathscr{P}$ is for the permutations of all particles. Then

$$\Psi = \zeta\mathscr{P}(\chi\chi)_0^{3K+1}\chi. \tag{6}$$

We first take the particles 1 and 2 into account, there are three cases: (i) one particle is alone (not in a pair) and the other one is in a pair, (ii) both in the same pair, and (iii) one in a pair and one in another pair. Accordingly, when 1 and 2 have been extracted, we have

$$\Psi \equiv \zeta(\Psi_I + \Psi_{II} + \Psi_{III}), \tag{7}$$

$$\Psi_I = 4(3K+1)\mathscr{P}_{12}\sum_{\lambda}\sqrt{2\lambda+1}w(3333;\lambda 0)[((\chi(1)\chi(2))_{\lambda}\chi]_3(\chi\chi)_0^{3K}, \quad (8)$$

$$\Psi_{II} = 2(3K+1)\mathscr{P}_{12}(\chi(1)\chi(2))_0(\chi\chi)_0^{3K}\chi, \quad (9)$$

$$\Psi_{III} = 12K(3K+1)\mathscr{P}_{12}\sum_{\lambda}(2\lambda+1)U\begin{pmatrix} 3,3,0 \\ 3,3,0 \\ \lambda,\lambda,0 \end{pmatrix}[((\chi(1)\chi(2))_{\lambda}(\chi\chi)_{\lambda}]_0(\chi\chi)_0^{3K-1}\chi, \quad (10)$$

where $\mathscr{P}_{12}$ denotes the permutations of all particles except 1 and 2, the W coefficient of Racah and the $9j$-Symbol for angular re-coupling have been introduced.

When $V_{12} = g_0 P_0^{12}$ acts on Ψ, only the terms with $\lambda = 0$ in Eqs.(8) and (10) can survive. Thus, we have

$$V_{12}\Psi = g_0\zeta\frac{(N-1)(N+6)}{7}\mathscr{P}_{12}(\chi(1)\chi(2))_0(\chi\chi)_0^{3K}\chi. \quad (11)$$

Obviously, a similar formula will be obtained for $V_{ij}\Psi$. Thereby

$$H\Psi = \frac{1}{2}\sum_{i\neq j}V_{ij}\Psi = g_0\zeta\frac{(N-1)(N+6)}{14}\mathscr{P}(\chi(1)\chi(2))_0(\chi\chi)_0^{3K}\chi = g_0\frac{(N-1)(N+6)}{14}\Psi. \quad (12)$$

Thus we conclude that Ψ is an exact eigen-state with the eigen-energy $E = g_0(N-1)(N+6)/14$. In this state the attractive effect of g_0 has been maximized.

Similarly, it can be proved that, for even N, $\Psi = \mathfrak{P}(\psi_3\psi_3)_0^K$ is also an exact eigen state with $E = g_0 N(N+5)/14$.
